# 

**Published:** 1883-04

**Authors:** 


| | PAGE. |
|---|---|
| **ORIGINAL COMMUNICATIONS:** | |
| Some Practical Remarks upon the Subject of Insanity. By Judson B. Andrews, M. D., | 385 |
| The Responsibility of the Examining Physician of the Insane, Particularly with Reference to their Commitment to Asylums. By P. M. Wise, M. D., | 403 |
| Hemorrhage from the Navel after Detachment of the Funis. By U. C. Lynde, M. D., | 411 |
| **FOREIGN CORRESPONDENCE,** | 413 |
| **SELECTIONS:** | |
| Changes of Uterine Muscular Tissue after Parturition, | 418 |
| **SOCIETY REPORTS:** | |
| Buffalo Medical and Surgical Association, | 418 |
| **EDITORIAL:** | |
| The Discussion of Ethics, | 422 |
| An Act to Regulate the Licensing of Practitioners of Physic or Surgery, | 424 |
| Obituary, | 428 |
| Editorial Notes, | 428 |
| **REVIEWS:** | |
| The Systematic Treatment of Nerve Prostration and Hysteria, | 430 |
| Transactions of the Medical Society of the State of Pennsylvania at its Thirty-third Annual Session, | 430 |
| The Physician Himself and What He Should Add to his Scientific Acquirements, | 430 |
| A Treatise on Fractures, | 431 |
| A Manual of Chemical Analysis as Applied to the Examination of Medicinal Chemicals, | 431 |
| Books Received, | 432 |

